# Prognostic Value of FDG-PET, Based on the Revised Response Criteria, in Patients with Malignant Lymphoma: A Comparison with CT/MRI Evaluations, Based on the International Working Group/Cotswolds Meeting Criteria

**Published:** 2015

**Authors:** Kayako Isohashi, Mitsuaki Tatsumi, Hiroki Kato, Kentaro Fukushima, Tetsuo Maeda, Tadashi Watabe, Eku Shimosegawa, Yuzuru Kanakura, Jun Hatazawa

**Affiliations:** 1 Department of Nuclear Medicine and Tracer Kinetics, Osaka University Graduate School of Medicine, Osaka, Japan; 2 Department of Radiology, Osaka University Graduate School of Medicine, Osaka, Japan; 3 Department of Hematology and Oncology, Osaka University Graduate School of Medicine, Osaka, Japan; 4 Department of Molecular Imaging in Medicine, Osaka University Graduate School of Medicine, Osaka, Japan; 5 Immunology Frontier Research Center, Osaka University, Osaka, Japan

**Keywords:** FDG-PET, CT, MRI, Malignant Lymphoma, Prognosis

## Abstract

**Objective(s):**

Post-treatment evaluations by CT/MRI (based on the International Working Group/Cotswolds meeting guidelines) and PET (based on Revised Response Criteria), were examined in terms of progression-free survival (PFS) in patients with malignant lymphoma (ML).

**Methods:**

79 patients, undergoing CT/MRI for the examination of suspected lesions and whole-body PET/CT before and after therapy, were included in the study during April 2007-January 2013. The relationship between post-treatment evaluations (CT/MRI and PET) and PFS during the follow-up period was examined, using Kaplan-Meier survival analysis. The patients were grouped according to the histological type into Hodgkin’s lymphoma (HL), diffuse large B-cell lymphoma (DLBCL), and other histological types. The association between post-treatment evaluations (PET or PET combined with CT/MRI) and PFS was examined separately. Moreover, the relationship between disease recurrence and serum soluble interleukin-2 receptor, lactic dehydrogenase, and C-reactive protein levels was evaluated before and after the treatment.

**Results:**

Patients with incomplete remission on both CT/MRI and PET had a significantly shorter PFS, compared to patients with complete remission on both CT/MRI and PET and those exhibiting incomplete remission on CT/MRI and complete remission on PET (P<0.001). Post-treatment PET evaluations were strongly correlated with patient outcomes in cases with HL or DLBCL (P<0.01) and other histological types (P<0.001). In patients with HL or DLBCL, incomplete remission on both CT/MRI and PET was associated with a significantly shorter PFS, compared to patients with complete remission on both CT/MRI and PET (P<0.05) and those showing incomplete remission on CT/MRI and complete remission on PET (P<0.01). In patients with other histological types, incomplete remission on both CT/MRI and PET was associated with a significantly shorter PFS, compared to cases with complete remission on both CT/MRI and PET (P<0.001). None of the serum parameters differed significantly between recurrent and non-recurrent cases.

**Conclusion:**

Post-treatment PET evaluations were well correlated with the outcomes of patients with ML, exhibiting FDG uptake. Among patients with HL or DLBCL, a post-treatment complete remission on PET was predictive of a relatively long PFS. For predicting the prognosis of patients with other histological types, a combination of CT/MRI and PET, rather than PET alone, is recommended.

## Introduction

The International Working Group (IWC) guidelines for response assessment in patients with non-Hodgkin’s lymphoma (NHL) and the Cotswolds Meeting (CMC) guidelines for patients with Hodgkin’s lymphoma (HL) are widely used (1, 2). Based on these guidelines, the size of malignant lymphoma (ML) lesions, which are visible on computed tomography (CT) scans, is the main basis for evaluations.

However, it is often difficult to differentiate post-treatment tumor tissues from the surrounding tissues on CT images due to the presence of necrotic and fibrous tissues. Consequently, the ability of CT scan to evaluate tumor response is somewhat limited and there is a possibility of underestimation (3).

The common types of ML including diffuse large B-cell lymphoma (DLBCL), follicular NHL, mantle cell lymphoma, and HL exhibit significant FDG uptakes. FDG-positron emission tomography (PET) is a well-established modality for the staging and monitoring of ML (4-13). FDG-PET frequently detects nodal and extranodal ML lesions that are missed using conventional imaging methods, and this may result in a more accurate staging of ML (4, 5, 12, 14). FDG-PET also allows viable tumor tissue to be distinguished from areas of necrosis or fibrosis in the residual mass after therapy; thus, FDG-PET is likely to provide more accurate and earlier evaluations compared to CT scan (3, 8, 15-18).

The Revised Response Criteria (RRC), which are mainly used to assess patient response in aggressive NHL and HL by incorporating FDG-PET findings, were first published in 2007 (15). Based on RRC, ML patients with negative FDG-PET results after the completion of therapy are considered to have entered the complete remission (CR).

In curable subtypes of lymphoma such as DLBCL and HL, accurate information on tumor status after treatment is essential (19). For incurable subtypes of lymphoma, objective response rate, CR rate (in particular) or progression-free survival (PFS) period are usually the primary endpoints of clinical trials, evaluating patient response to treatment (19, 20).

In this study, we retrospectively investigated post-treatment CT/magnetic resonance imaging (MRI) evaluations, based on IWC/CMC guidelines and PET evaluations, based on RRC in ML patients. We also examined the relationship between these evaluations and PFS during the follow-up period.

Moreover, serum soluble interleukin-2 receptor (sIL-2R), serum lactic dehydrogenase (LDH), and C-reactive protein (CRP) have been recently used as markers of tumor burden and disease activity in ML. In fact, sIL-2R and LDH are significant prognostic indicators in NHL (21-23), and serum sIL-2R and CRP levels are helpful prognostic indices in patients with HL (24). Therefore, we also examined the association between these tumor markers (i.e., sIL-2R, LDH, and CRP) before and after treatment and disease recurrence.

## Methods

### 

#### Patients

A total of 89 patients with ML underwent whole-body FDG-PET before and after treatment and were evaluated, based on RRC during April 2007-January 2013 at our institute.

In total, 10 out of 89 patients were excluded from the study due to the following reasons: not being an adult (≥20 years old) (one patient), absence of a significantly abnormal FDG accumulation on PET evaluation before the treatment (one patient), primary cancer accompanied by ML (three patients), diabetes mellitus (one patient), high blood glucose level (>150 mg/dl) immediately before FDG injection (two patients), and change of hospital without follow-up (for at least 6 months) after treatment at our hospital (two patients). Young patients were excluded since pediatric lymphoma differs from adult lymphoma in terms of form, treatment response, and patient prognosis (25, 26).

The remaining 79 patients (42 men and 37 women; median age: 58 years; range: 20-83 years) were analyzed. Overall, 14 patients had HL and 65 cases had NHL. The diagnosis of ML was confirmed histopathologically in all patients. The patients’ characteristics are listed in (Table 1 and 2).

**Table 1:** Staging of patients and histological characteristics of the disease

Initial stage (Ann Arbor)	I: 13, II: 17, III: 14, IV: 35
Histology	
Hodgkin’s lymphoma	14
Diffuse large B-cell lymphoma	31
Follicular lymphoma	21
Mucosa-associated lymphoid tissue lymphoma	4
Lymphoplasmacytic lymphoma	3
Marginal zone B-cell lymphoma	1
Anaplastic large cell lymphoma	1
Mantle cell lymphoma	1
Natural killer T-cell lymphoma	1
Intravascular lymphoma	1
Burkitt lymphoma	1

**Table 2:** Treatment characteristics of patients

Therapy	Number
Chemotherapy and Rituximab	46
Chemotherapy and radiotherapy	6
Chemotherapy	12
Rituximab	9
Chemotherapy, radiotherapy and Rituximab	4
Surgical therapy and Rituximab	2
Surgical therapy and chemotherapy	1
Surgical therapy and radiotherapy	1
Surgical therapy, chemotherapy and Rituximab	1

Three patients experienced disease recurrence during the study period. These cases were successfully treated, and post-treatment evaluations were performed twice for each of these patients. This study was performed after obtaining the approval of the institutional ethics committee for clinical research at Osaka University.

#### Imaging protocols

All patients underwent whole-body FDG-PET/CT and CT/MRI examinations for suspected lesions before and after therapy. Post-treatment FDG-PET/CT scans were performed at least three weeks after the completion of chemotherapy and 8 weeks after the completion of radiation or chemoradiotherapy, according to RRC (15). Cases not complying with these criteria were excluded from the study, even if treated.

Whole-body FDG-PET/CT was performed, using a Gemini GXL PET/CT system (Philips, Cleveland, OH, USA) or an Eminence PET/CT system (Shimadzu, Kyoto, Japan) during 2007-2013. After fasting for at least four hours, patients received an intravenous injection of FDG (3.7 MBq/kg for PET/CT) and images were acquired 60 min later. FDG-PET/CT scans were obtained from the parietal to mid-femur level.

#### Imaging interpretation and analysis

All FDG-PET/CT and CT/MRI scans were performed at the same nuclear medicine/radiology department and were reviewed by three radiologists and nuclear medicine physicians (K.I., H.K. and J.H.) on the same team; the reviewers were blinded to patient outcomes. Any equivocal cases were resolved by consensus.

The size of each nodal mass on CT/MRI images was measured, based on two-dimensional diameters in the transverse plane before and after therapy. The sum of the product of diameters was evaluated for each mass, using IWC guidelines for NHL and CMC guidelines for HL (1, 2).

On PET images, clear foci of increased FDG uptake over the background, not located in areas of normal FDG uptake and/or excretion, were considered positive for tumor cells and were evaluated, based on RRC as follows (15): CR, defined as no evidence of PET-positive disease before therapy; partial remission (PR), defined as reduced evidence of PET-positive disease before therapy and absence of new sites; stable disease (SD), known as failure to attain CR/PR, without the progressive disease (PD); and PD, defined as the appearance of new PET-positive lesions or increased abnormal FDG uptake in previously involved site.

Post-treatment CT/MRI (based on IWC/CMC guidelines) and PET evaluations (based on RRC) were investigated in relation to PFS during the follow-up period. PFS was calculated since the completion of therapy until disease progression, the last follow-up session, or hospital transfer. For cases with hospital transfer, follow-up for at least six months after the completion of therapy was performed at our hospital prior to transfer.

PET scans are recommended for response assessment in patients with HL or DLBCL (15, 19). All patients were grouped according to the histological type as follows: HL or DLBCL, and other histological types. PET evaluations and PET combined with CT/MRI were also investigated regarding PFS during the follow-up period, respectively.

#### Laboratory data analysis

Most patients underwent routine evaluations including physical examinations and laboratory studies such as the measurement of serum sIL-2R, LDH, and CRP levels as tumor markers within 14 days before the start of therapy (pre-treatment) and after the completion of therapy (post-treatment).

Serum sIL-2R level (normal range: 127-582 U/ml) was measured, using a sandwich enzyme immunoassay. The serum LDH level (normal range: 103-229 U/L) was determined, using a method described by the Japan Society of Clinical Chemistry, using lactic acid as the substrate; the serum CRP level (normal <0.2 mg/dl) was measured, using latex immune nephelometry.

During the follow-up period after the end of treatment, patients diagnosed with disease recurrence or disease progression, based on physical examinations and follow-up images, were included in the recurrent group. The association between tumor marker levels before and after treatment and disease recurrence was also examined.

#### Statistical analysis

PFS was compared between the groups, using log-rank test. The comparison between each parameter (i.e., sIL-2R, LDH, and CRP) before and after treatment between the recurrent and non-recurrent groups was performed, using Mann-Whitney U test. For all statistical analyses, P-value < 0.05 was considered statistically significant. All the analyses were performed, using Stat Mate IV (ATMS Co., Ltd., Tokyo, Japan).

## Results

A total of 82 post-treatment evaluations in 79 ML patients were analyzed. The median follow-up duration was 45 months (range: 4-88 months). The results of post-treatment CT/MRI and PET evaluations were consistent in 78% of cases (64:82). CR on both CT/MRI and PET was the most common status (62%, 51:82). Among the CT/MRI and PET evaluations with results that were not concordant, 94% (17/18) had incomplete remission on CT/MRI and complete remission on PET. These results are presented in (Table 3).

**Table 3:** Post-treatment evaluations, using CT/MRI and PET (n=82)

	CR on PET	PR on PET	SD on PET	PD on PET
CR on CT/MRI	51	0	0	0
PR on CT/MRI	17	10	0	0
SD on CT/MRI	0	1	1	0
PD on CT/MRI	0	0	0	2

Abbreviations: CR, complete remission; PR, partial remission; PD, progressive disease; SD, stable disease

**Figure 1:**
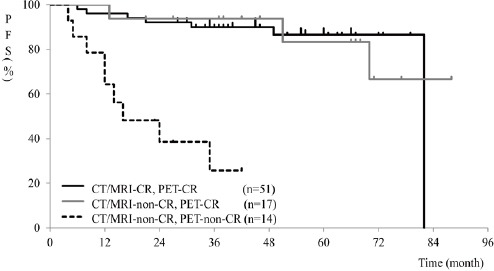
Kaplan-Meier curves for PFS in different settings

The 3-year PFS rates in cases with CR on both CT/MRI and PET (n=51), incomplete remission on CT/MRI and CR on PET (n=17), and incomplete remission on both CT/MRI and PET (n=14) were 90%, 94%, and 26%, respectively. Patients with incomplete remission on both CT/MRI and PET had a significantly shorter PFS, compared to patients with CR on both CT/MRI and PET (*P*<0.001) and those exhibiting incomplete remission on CT/MRI and CR on PET (*P*<0.001) (Figure 1). No significant difference was observed between cases with CR on both CT/MRI and PET and those with incomplete remission on CT/MRI and CR on PET (*P*=0.984).

**Figure 2:**
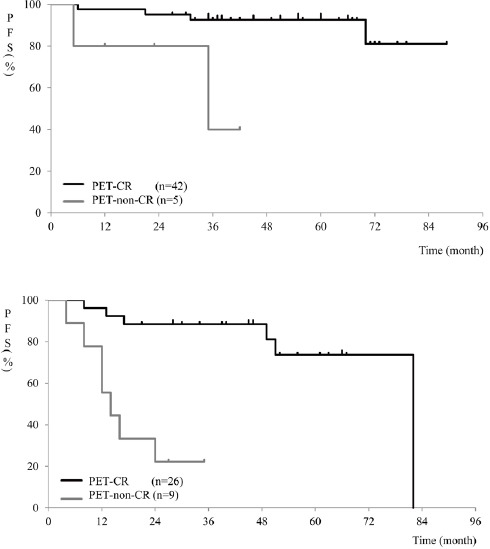
Comparison of PFS curves in patients with CR on PET versus those with incomplete remission on PET in HL or DLBCL (A) and other histological subtypes (B)

As the RRC has recommended, patient response was assessed, using PET in patients with HL and DLBCL. These cases were divided into two groups (HL or DLBCL, and other histological types) and the following results were obtained. Among patients with HL or DLBCL, those with CR on PET had a longer PFS, compared to patients with incomplete remission on PET (*P*<0.01) (Figure 2A). Among patients with other histological types, cases with CR on PET also had a longer PFS, compared to those with incomplete remission on PET (*P*<0.001) (Figure 2B).

**Figure 3:**
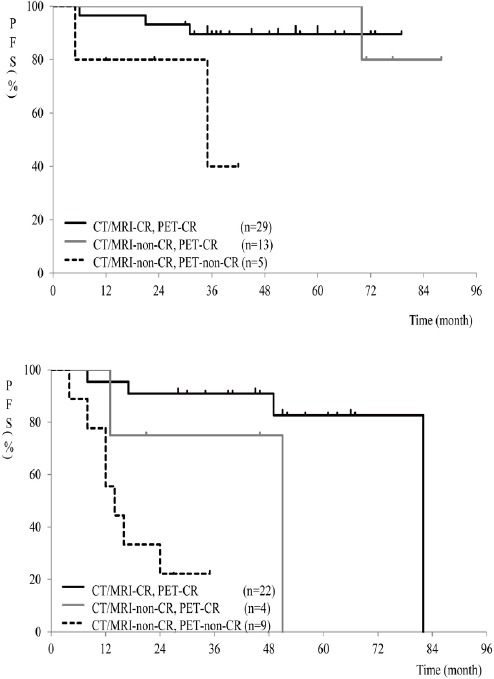
Comparison of PFS curves in different settings in HL or DLBCL (A) and other histological subtypes (B)

Further evaluation of combined CT/MRI and PET in patients with HL or DLBCL showed that incomplete remission on both CT/MRI and PET was associated with a significantly shorter PFS, compared to cases with CR on both CT/MRI and PET (*P*<0.05) and those exhibiting incomplete remission on CT/MRI and CR on PET (*P*<0.01) (Figure 3A). No significant difference was observed between cases with CR on both CT/MRI and PET and those showing incomplete remission on CT/MRI and CR on PET (*P*=0.568).

Among patients with other histological types, incomplete remission on both CT/MRI and PET was associated with a significantly shorter PFS, compared to patients with CR on both CT/MRI and PET (*P*<0.001) (Figure 3B). Although no significant difference was observed between cases with CR on both CT/MRI and PET, compared to those with incomplete remission on CT/MRI and CR on PET, the former group tended to have a longer PFS than the latter (*P*=0.058). No significant difference was observed between cases with incomplete remission on CT/MRI and CR on PET and those showing incomplete remission on both CT/MRI and PET (*P*=0.617).

Disease recurrence occurred in 18 out of 82 cases, who were evaluated during the follow-up period. None of serum parameters (sIL-2R, LDH, and CRP) differed significantly between recurrent and non-recurrent cases (Table 4).

**Table 4:** Comparison of pre- and post-treatment serum parameters and disease recurrence

Value	Evaluation time (number)	Recurrent group	Non-recurrent group	*P*-value
SIL-2R (U/ml)	Pre (n=80)	1872 ± 2167	2293 ± 3096	0.715
	Post (n=74)	576 ± 297	434 ± 187	0.166
LDH (U/L)	Pre (n=82)	222 ± 104	294 ± 300	0.311
	Post (n=79)	218 ± 87	212 ± 56	0.900
CRP (mg/dl)	Pre (n=82)	0.36 ± 0.97	0.93 ± 2.1	0.066
	Post (n=81)	0.26 ± 0.44	0.27 ± 0.73	0.981

Abbreviations: Pre = pre-treatment, Post= post-treatment, SD = standard deviation

Data are shown as mean±SD.

## Discussion

This study showed that cases with post-treatment CR on both CT/MRI and PET and subjects with incomplete remission on CT/MRI and CR on PET had a longer PFS, compared to ML cases with incomplete remission on both CT/MRI and PET. This study also showed that post-treatment PET evaluations were well correlated with the outcomes of patients with HL or NHL, exhibiting FDG uptake.

Even if post-treatment CT/MRI and PET results differed, post-treatment CR on PET was predictive of a long PFS among patients with HL or DLBCL. Among patients with other histological types, an evaluation consisting of a combination of CT/MRI and PET, rather than PET alone, is recommended.

Many ML subtypes have high cell density and exhibit high FDG uptake. In particular, aggressive types such as DLBCL and lymphoblastic lymphoma exhibit relatively high FDG uptakes (27). HL is composed of a few scattered neoplastic cells, referred to as Hodgkin and Reed-Sternberg cells, which are surrounded by mononuclear cells with a high metabolic activity (13). To evaluate patient response to treatment, FDG-PET is recommended for patients with aggressive NHL or HL, given the consistent FDG avidity of these lesions and their potential curability (11, 15).

Unlike CT and MRI, FDG-PET can detect metabolic changes earlier than morphological changes after treatment induction and can distinguish the remaining viable tumor tissues from inactive scar tissues after treatment (11, 14, 27, 28). A meta-analysis by Zijlstra et al. showed that the pooled sensitivities and specificities of FDG-PET for evaluating patient response to first-line therapy were 72% and 100% for NHL and 84% and 90% for HL, respectively (29). Our study also showed that regardless of CT/MRI evaluation, post-treatment PET evaluation is well correlated with the outcomes of patients with HL or DLBCL.

However, PET is limited in its ability to detect microscopic lesions, considering the partial volume effect (PVE). Since PVE is influenced by factors including tumor size and shape, tumor shrinkage after treatment tends to be underestimated in some cases (30, 31). Therefore, patient response evaluations, based on PET findings, are associated with a risk of failure to predict late relapse (29).

About two-thirds of patients with HL and one-third of patients with NHL have residual masses visible on CT scan. They have negative PET results after the completion of therapy and clinical or other biochemical signs of relapse are absent in these cases; however, relapse occurs in less than 10% of patients with HL and 15-20% of patients with NHL (27).

In this study, 17 cases with CR on PET exhibited a residual mass lesion, based on CT/MRI examinations; recurrence was confirmed in three cases during the follow-up after the end of treatment. Although no statistically significant difference was found between patients with CR on both CT/MRI and PET and those exhibiting incomplete remission on CT/MRI and CR on PET, subjects with morphological changes, accompanied by metabolic alterations after therapy, tended to have a longer PFS; this tendency was particularly observed in patients with histological types other than HL and DLBCL.

In this study, among 34 cases with other histological types, the most frequent diagnosis was follicular NHL (21 cases). Among studies focusing on PET evaluations, several reports have indicated that a negative PET status at the end of the treatment is associated with a longer PFS, compared to a PET-positive status in patients with follicular NHL (32-34). The present study also showed that post-treatment CR on PET was predictive of a prolonged CR duration among patients with other histological types.

On the other hand, although a statistically significant difference was not observed, patients with CR on both CT/MRI and PET tended to have a longer PFS, compared to those with incomplete remission on CT/MRI and CR on PET.

Follicular lymphoma is an incurable lymphoma subtype. The relapse rate among patients with follicular lymphoma is higher than that of patients with other more aggressive lymphomas (35). A reduction in the number of viable tumor cells can lead to a decrease in the anatomical tumor size (36). Therefore, the combination of CT/MRI and PET is likely to be more advantageous than PET alone for predicting the prognosis of patients with other histological types.

In the current study, none of the examined serum markers were capable of predicting disease recurrence. Standardized uptake values on PET images may be a better marker of disease status in patients with NHL, compared to sIL-2R level (37). The advantage of imaging for the prediction of disease recurrence was also evident in the current study, which examined a relatively large number of cases and histological types and had a median follow-up duration of 45 months.

The present study had a few limitations. Firstly, the study population was comprised of cases with different HL and NHL subtypes, and the number of cases with some subtypes was low. Secondly, not all lesions with abnormal FDG uptake were histopathologically examined.

## Conclusion

In conclusion, post-treatment CR on both CT/MRI and PET was predictive of a long PFS in patients with ML. Post-treatment PET evaluations were well correlated with the outcomes of patients with ML, exhibiting FDG uptake. Among patients with HL or DLBCL, post-treatment CR on PET was predictive of a relatively long PFS. When predicting the prognosis of patients with other histological types, a combination of CT/MRI and PET, rather than PET alone, is recommended.
